# The impact of employment programs on common mental disorders: A systematic review

**DOI:** 10.1177/00207640221104684

**Published:** 2022-07-07

**Authors:** Libby Evans, Crick Lund, Alessandro Massazza, Hannah Weir, Daniela C Fuhr

**Affiliations:** 1 Department of Health Services Research and Policy, London School of Hygiene and Tropical Medicine, UK; 2 Centre for Global Mental Health, Health Services and Population Research Department, Institute of Psychiatry, Psychology and Neuroscience, King’s College London, UK; 3 Department of Psychiatry and Mental Health, Alan J. Fisher Centre for Public Mental Health, University of Cape Town, South Africa

**Keywords:** Employment programs, common mental disorders, poverty, systematic review, mental health, depression, anxiety

## Abstract

**Background:**

While employment programs were not created with the intent to improve common mental disorders (CMDs), they may have a positive impact on the prevalence, incidence, and severity of CMD by reducing poverty and increasing access to economic mobility.

**Aim:**

To examine and synthesize the available quantitative evidence of the impact of employment programs on outcomes of CMD.

**Methods:**

Embase, Econlit, Global Health, MEDLINE, APA PsychINFO, and Social Policy and Practice were searched for experimental and quasi-experimental studies which investigated the impact of employment programs on primary and secondary outcomes of a CMD. A narrative synthesis according to Popay was conducted. The methodological quality of studies was assessed with the Cochrane Risk of Bias tool and the Newcastle-Ottawa Assessment Scale.

**Results:**

Of the 1,327 studies retrieved, two randomized controlled trials, one retrospective cohort, one pilot study with a non-randomized experimental design, and one randomized field experiment were included in the final review. Employment programs generally included multiple components such as skills-based training, and hands-on placements. Depression and anxiety were the CMDs measured as primary or secondary outcomes within included studies. Findings regarding the impact of employment programs on CMD were mixed with two studies reporting significantly positive effects, two reporting no effects, and one reporting mixed effects. The quality among included studies was good overall with some concerns regarding internal validity.

**Conclusion:**

Employment programs may support a decrease in the prevalence, incidence, and severity of CMDs. However, there is high heterogeneity among study effects, designs, and contexts. More research is needed to gain further insight into the nature of this association and the mechanisms of impact. This review highlights the potential for employment programs and other poverty-reduction interventions to be utilized and integrated into the wider care, prevention, and treatment of common-mental disorders.

## Introduction

Globally, mental disorders are the largest contributor to the global burden of disease (GBD) and to years lived with disability (YLD; Rehm & Shield, 2019; Vigo et al., 2016). As of 2018, common mental disorders (CMDs) which include depression and anxiety, accounted for 12.7% of total YLDs and made up 5.6% of disability adjusted life years (DALYs; James et al., 2018). Treatment quality and coverage for CMDs varies drastically based on context, disorder, and availability of resources (World Health Organization [WHO], 2017). While traditional psychosocial and/or psychotherapy interventions have had moderate effects in reducing individual symptomology (Alegria et al., 2021) these treatments often fail to adequately address the social determinants of mental illness (Burgess et al., 2020; Davaasambuu et al., 2020) that may help in supporting the long term recovery of mental health problems. Social determinants of mental illness are understood as societal factors that can influence the prevalence, incidence, and severity of a mental disorder with poverty being one of the determinants that is most strongly associated with poor mental health (Alegria et al., 2018). Indeed, people living in poverty suffer from increased prevalence, severity, duration, and worse outcomes for CMDs (Lund & Cois, 2018; Lund et al., 2010; Patel & Kleinman, 2003; Ridley et al., 2020). Research has also shown that multiple aspects of poverty, such as food insecurity, low socio-economic status, limited education, and financial stress, all have a consistently strong positive association with CMDs (Lund et al., 2010).

CMDs are also strongly associated with unemployment (Lund et al., 2010; Ridley et al., 2020; Wahlbeck et al., 2017). Research shows that those who are unemployed are twice as likely to experience major depressive disorder (MDD) or depressive symptoms and not having a way to create sufficient money for the livelihood of oneself and one’s family is associated with an increase in perceived stress and hopelessness (Dooley et al., 1994; Lund, et al., 2011; Patel & Kleinman, 2003). Simultaneously, people with mental health problems have also been found to be disproportionately affected by barriers to economic freedom caused by unemployment with prevalence of mental illness being associated with reduced employment rates (Lund et al., 2010; Ridley et al., 2020).

Because poverty, inequality, and CMDs are closely interlinked, interventions that seek address social determinants can have a positive impact on mental health (Silva et al., 2016; WHO, 2008). Focusing on creating opportunities for the expansion of freedom through poverty reduction and access to employment may also be vital for preventative mental health care (Sen, 2014). Employment programs are one example of interventions that can address the social determinants of poor mental health and increase access to social and economic capital (Alegria et al., 2018).

The purpose of employment programs is to create employment engagement for participants (International Labour Organization, 2021.), whether this is through the more common public work program – such as traditional government programs providing employment opportunities to low income families – or relatively newer programs such as skills-based training, work placements, or food-for-work programs (International Labour Organization, 2021.) Employment programs increase economic mobility and can reduce poverty by providing participants with the skills, experience, or resources necessary to aid in securing consistent income-generating activities (International Labour Organization, 2021.; Wahlbeck et al., 2017). However not all employment programs provide participants with direct employment; some may provide entrepreneurial training, or aid in the creation of entrepreneurial endeavors such as agribusiness and craftsmanship as a form of poverty reduction (International Labour Organization, 2021). While many employment programs are not created with the intent to prevent or treat CMDs, by addressing poverty, financial stress, and other social determinants of CMDs a reduction in prevalence, incidence, and severity of these disorders may occur.

High income countries (HIC) have successfully utilized vocational rehabilitation and other comparable employment programs to target poverty and unemployment among those suffering from disability (Chimara et al., 2021; Marshall et al., 2014). Vocational rehabilitation often focuses on decreasing barriers to employment for those with severe disabilities by providing the therapy, rehabilitation, and/or training necessary for such individuals to obtain employment (Chamberlain et al., 2009). Supported employment is one intervention that has been implemented rigorously in the United States and other HICs. Conversely, the emphasis of employment programs in low- and middle-income countries (LMIC) is often less on engaging participants via train-and-place model or traditional vocational rehabilitation programs such as supported employment and more focused on entrepreneurial endeavors or microfinance groups aimed at increasing household revenue, resources, and economic capital (Mcguire et al., 2020; Owusu-Addo et al., 2018; Wahlbeck et al., 2017).

Previous reviews have found more traditional employment programs to be effective in supporting the recovery of mental illness. For example, supported employment, and more specifically individual placement support (IPS) has been extensively researched and found to decrease psychiatric symptoms and increase employment rates among those with SMI in multiple contexts (Marshall et al., 2014; Modini et al., 2016). IPS and similar supportive programs focus on placing people living with SMI into employment and then supporting them in maintaining that employment; taking a ‘place and train’ model over the more traditional ‘train and place’ models (Modini et al., 2016). However, most studies regarding employment programs assess outcomes relating to employment rates and the number of people placed in successful employment rather than mental health.

Reviews have been conducted regarding the impact of social protection programs, such as conditional and unconditional cash transfers, on both physical and mental health in LMICs but with focus on SMI and CMDs (Davaasambuu et al., 2020; Lagarde et al., n.d.; Lorenzetti et al., 2017; Mcguire et al., 2020; Omalley & Burke, 2017; Owusu-Addo & Cross, 2014; Owusu-Addo et al., 2018; Ranganathan & Lagarde, 2012; Zimmerman et al., 2021). Such programs have been found to be effective in reducing poverty and breaking down structural barriers to health (Owusu-Addo et al., 2018).

However, little is currently known about the impact of more traditional employment programs targeting general populations on CMD outcomes. In recent years, many studies have included mental health as primary or secondary outcomes in their evaluation of economic and wider health benefits of employment programs. Nonetheless, to the best of our knowledge there is no study that synthesized the existing research regarding the effect of these programs on CMDs. This study will systematically review existing literature on the impact of employment programs on CMDs. More specifically the objectives of this systematic review are to:

1. To synthesize existing evidence on the impact of employment programs on outcomes of CMDs.
2. To assess the overall quality of studies.
3. To identify current gaps in research and make recommendations for future research, policy, and care initiatives.

## Methods

This review was conducted following the Preferred Reporting Items for Systematic reviews and Meta-Analysis (PRISMA) guidelines 2020 version (Page et al., 2021). The protocol was registered on PROSPERO (registration number: CRD42021230930).

### Search strategy

Peer-reviewed articles from the following databases were searched from inception until December 2021: Econlit, Embase, Global Health, MEDLINE, APA PsycINFO, and Social Policy and Practice.

In contrast to previous studies which focused on employment outcomes, the primary outcome of focus within this review was depression, anxiety, somatoform disorders, or other CMDs such as somatoform disorders among those who participated in an employment program as defined above. Studies with participants of all ages, nationalities, and settings were included. Interventions included employment programs such as skills-based training, supported employment, job-matching/job-placement assistance, and public work programs. Many of the screened studies focused on return-to-work programs for those on sick leave and/or psychological rehabilitation as a vocational rehabilitation tool. Such vocational rehabilitation studies were only included if the intervention had some form of active employment component such as job training, skills building, or job placement assistance. Rehabilitation interventions could include return to work or psychosocial components but must have an active employment component integrated within the program to be eligible for inclusion. Experimental and quasi-experimental studies and mixed method study designs were included.

Studies with participants who had been diagnosed with a SMI, alcohol use disorder, or autism were excluded. Studies with interventions which provided only microfinance, microcredit, or cash loans without an employment component were excluded. Experimental and quasi-experimental studies and mixed method study designs were included. Gray literature was not searched or included in this review. All non-English studies were excluded. Supplemental Appendix 2 includes more information on inclusion and exclusion criteria.

The search strategy combined terms for common mental disorders (depression, anxiety, and somatoform disorders) with employment program terms (entrepreneur, job-match, skills building, cash-transfer, microfinance, microcredit, vocational rehabilitation, occupational rehabilitation, work program, job placement assistance, and cash grant; search strategy included in Supplemental Appendix 1).

### Data extraction and synthesis

Data was extracted by one reviewer using a data extraction form within Covidence software. Data was synthesized narratively according to guidelines of Popay et al. (2006).

### Quality appraisal

Randomized control trials were assessed with the Cochrane Risk of Bias (RoB2) tool. The Newcastle-Ottawa Assessment Scale was used for quality assessment of all included case-control and cohort studies.

## Results

**Figure 1.**
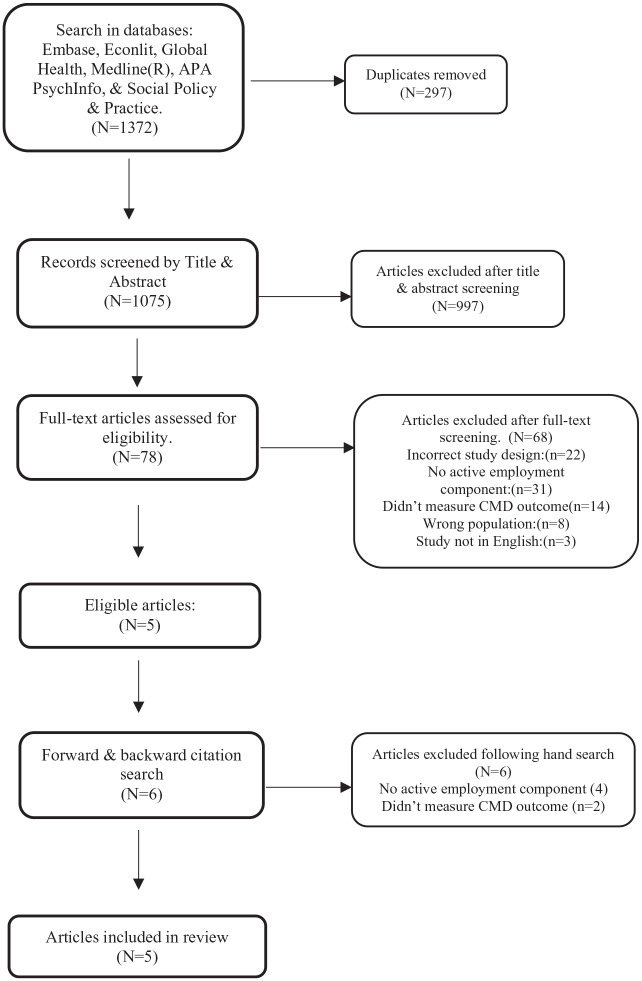
PRISMA flowchart.

The article selection process is summarized below in Figure 1.

### Study characteristics

Information on individual studies is shown in Table 1. Date of publication ranged between 1985 and 2021. Of 5 included studies, 2 (40%) were from LMICs, with 1 from both Bangladesh (Karasz et al., 2021) and the Democratic Republic of the Congo (Glass et al., 2017). Three studies were from HIC, two of which were from the United States of America (Bellotti et al., 2011; Vinokur et al., 2000), and one from the United Kingdom (Branthwaite & Garcia, 1985). Sample size was small in some studies, with only 2 (40%) having a sample size of over 200 participants. Three studies had smaller sample sizes of under 100 (Bellotti et al., 2011; Branthwaite & Garcia, 1985; Karasz et al., 2021).

**Table 1.** Information extracted from individual studies.

Article reference	Country	Study design	Sample size	*M*_age_	Participants	Intervention	Time to follow-up	CMD measurement	Primary or secondary outcome	Results
Glass et al. (2017)	Democratic Republic of Congo	RCT	833	25+	Families living in area with historical conflict and human rights violations in South Kivu province.	‘Pigs for Peace’ productive asset ‘credit’ transfer of a female piglet aged 2–4 months conditional on building a pig pen, and giving two piglets from initial litter as repayment. Family can then use livestock and subsequent litters how they choose. Also receive skills training on livestock nutrition and care, as well as basic health services for the livestock.	Follow-up 18 months after receiving ‘transfer’.	Hopkins Symptom Checklist (HSCL)	Primary	Those who received intervention had significant improvement in anxiety (*d* = 0.15, *p* < .05) but not depression (*d* = 0.11, *p* = 0.89).
Karasz et al. (2021)	Bangladesh	Pilot RCT	48	26	Housewives with depressive symptoms living in rural Bangladesh.	12 group sessions (8 depression treatment and 4 financial literacy education followed by a cash transfer). At beginning of project each opened a bank account and made regular deposits of approximately $2.5/month. Participants were given the option of engaging in income-producing activities, such as tree planting, to earn small amounts of cash to make deposits. At the 12 months point, participants were given an up to 6× match of their savings, with a maximum total of $186 (equivalent to the cost of two goats).	Follow-up 12 months after receiving intervention.	Patient Health Questionnaire − 9	Primary	Intervention group had a significant decrease in depression compared to the control group (β = −.8, *p* < 0.001)
Bellotti et al. (2011)	USA	Pilot non-randomized experimental study	19	35	Students in the Veterans conservation corps at Green River community college in Auburn, Washington.	Training program at Green River Community College consisting of 3 days a week in educational classes, 2 days in the field learning hands on skills. $1,000 monthly stipend	5 months between baseline and midline. About 10 months between baseline and endline.	Beck Depression Inventory and Beck Anxiety Inventory	Primary	No significant changes in either depression or anxiety measures. (*p* > .05)
Branthwaite and Garcia (1985)	United Kingdom	Mixed methods, retrospective cohort.	46	17	Young people in the UK either employed as apprentices, on a placement scheme, on a project scheme, or unemployed.	2 types of training schemes. Placement schemes where the participant is placed to work within a company and project schemes where groups of young people work under a supervisor on a project in fields such as decorating, carpentry, bricklaying, or tree-planting.	Follow-up differed for different groups. For those in project and placement schemes measurement occurred between 1 week and 12 months after receiving intervention.	Beck Depression Inventory	Primary	Those in the intervention groups were not significantly less depressed than the unemployed group (apprentices vs. unemployed: *U* = 15.5, *p* < .025). They were significantly more depressed (apprentices vs. placement scheme: *U* = 74, *p* < .025; apprentices vs. project scheme: *U* = 28, *p* < .05) than the employed apprentices.
Vinokur et al. (2000)	USA	RCT	1,801	36.2	Recently unemployed job seekers.	Five, 4 hours sessions over a 1 week period. Intended to increase sense of mastery and motivation to search for a job by learning job-search skills and inoculation against setbacks.	2 years following initial intervention implementation.	Short version of composite international diagnostic interview and 11 items based on Hopkins Symptom Checklist.	Primary	Compared with those in the control group, intervention participants had significantly lower levels of depressive symptom (*d* = −0.06, *p* < .05) and were significantly less likely to meet criteria for a major depressive episode (*d* = −0.49, *p* < .05).

Adults were the target population in all studies with the mean age of participants ranging from 17 to 36 (Bellotti et al., 2011; Branthwaite & Garcia, 1985; Glass et al., 2017; Karasz et al., 2021; Vinokur et al., 2000). One of the interventions targeted women or had samples that were more than 75% female (Karasz et al., 2021). Most participants were not depressed at time of entry into the studies. Only two studies (18%) measured CMD prior to screening (Karasz et al., 2021; Vinokur et al., 2000), meaning most studies were looking at the impact of employment programs on the general population’s rate of CMD and not the impact of programs on those with a current clinical diagnosis of a CMD.

Included studies had a range of study designs. Three (50%) were randomized control trials (RCT; Glass et al., 2017; Karasz et al., 2021; Vinokur et al., 2000) with one retrospective cohort design (Branthwaite & Garcia, 1985), one pilot study with a non-randomized experimental design (Bellotti et al., 2011).

### Intervention characteristics

Many of the interventions involved multiple components aiming to target different aspects of unemployment, poverty, and/or psychosocial wellbeing. There were eight different active employment programs among the five included studies. Microcredit was a common supplement within interventions with three including some form of microfinance or cash transfer component in the intervention (Bellotti et al., 2011; Glass et al., 2017; Karasz et al., 2021). All five studies included a skills-based training component (Bellotti et al., 2011; Branthwaite & Garcia, 1985; Glass et al., 2017; Karasz et al., 2021; Vinokur et al., 2000). Skills taught in skills-based training interventions included job-search skills, livestock care and agribusiness, craftsmanship, education, entrepreneurship, and financial management/literacy. Further information on the skills-based training components for relevant studies can be found in Supplemental Appendix 3. Finally, two studies utilized hands-on work placements – where participants were placed in a position and learned hands-on how to do the work (Bellotti et al., 2011; Branthwaite & Garcia, 1985).

For interventions with multiple components, 2 (40%) had both skills-training and placement components (Bellotti et al., 2011; Branthwaite & Garcia, 1985). While most interventions simply focused on different employment components, one intervention included psychoeducation group sessions alongside a matched savings program and financial literacy group sessions (Karasz et al., 2021).

### Outcome measurement

Only depression and anxiety were measured as outcomes in included studies. Depression was the most prevalent CMD outcome measure with all five studies (Bellotti et al., 2011; Branthwaite & Garcia, 1985; Glass et al., 2017; Karasz et al., 2021; Vinokur et al., 2000;) including it as a primary outcome. Instruments for measuring depression varied, with two studies utilizing Beck’s Depression Inventory (Bellotti et al., 2011; Branthwaite & Garcia, 1985), two Hopkins Symptom Checklist (Glass et al., 2017; Vinokur et al., 2000), one Short version of Composite International Diagnostic Interview (Vinokur et al., 2000), and one Patient-Health Questionnaire − 9 (Karasz et al., 2021). Anxiety was only measured as a primary outcome in two of the five studies (40%; Bellotti et al., 2011; Glass et al., 2017). Instruments used to measure anxiety included Beck’s Anxiety Inventory (Bellotti et al., 2011) and the Hopkins Symptom Checklist (Glass et al., 2017).

High heterogeneity existed within the scales used to measure both depression and anxiety. Multiple versions and cut-off points were used within studies that used the same measures, making it difficult to directly compare study outcomes.

Measurement of outcomes also occurred at different timepoints, ranging from current enrollment to 2 years following program completion. Different follow-up times were used in different studies, making direct comparison between studies challenging.

### Impact on CMDs

Of the 5 studies that measured depression as a primary or secondary outcome, 2 (40%) found a significant positive effect (Karasz et al., 2021; Vinokur et al., 2000) and 3 (60%) found no effect (Bellotti et al., 2011; Branthwaite & Garcia, 1985; Glass et al., 2017;). Within the 2 that measured anxiety, 1 (50%) found a positive effect (Glass et al., 2017), 1 (50%) no effect (Bellotti et al., 2011).

The different employment components yielded conflicting results on CMDs. Of the interventions with a skill-based component, 3/5 (60%) had a significant positive effect on CMD outcome measures (Karasz et al., 2021; Vinokur et al., 2000), while 2/5 (40%) had no effect (Bellotti et al., 2011; Branthwaite & Garcia, 1985). Both interventions with a placement and a skills-based training component also found no effect on mental health (Bellotti et al., 2011; Branthwaite & Garcia, 1985).

All studies with an RCT design (3/3) reported a positive impact on at least one CMD (Glass et al., 2017; Karasz et al., 2021; Vinokur et al., 2000). Effect sizes for the Glass et al. (2017) study were *d* = 0.15, *p* < .05 for anxiety and *d* = 0.11, *p* = .89 for depression; The Karasz et al. (2021) RCT reported and effect size of β = −.8, *p* < .001. Similarly, Vinokur et al. (2000) experiment also found positive effects, reporting effect sizes of *d* = −0.06, *p* < .05 in comparing depressive symptoms between the control and intervention groups and *d* = .49, *p* < .05 for the reporting of a Major Depressive Episode. Studies with retrospective, and non-randomized experimental designs all reported null effects (Bellotti et al., 2011; Branthwaite & Garcia, 1985). Bellotti et al. (2011) found no significant changes in either depression or anxiety measures (*p* > .05) while Branthwaite and Garcia (1985) reported no difference in depression among those receiving the intervention and the control group (apprentices vs. unemployed: *U* = 15.5, *p* < .025).

### Quality appraisal

Risk of bias in RCT studies was judged to have some concerns overall (see Supplemental Appendix 4 for more information) around allocation concealment, blinding of participants and personnel, and incomplete outcome data. Both cohort studies were judged to have high risk of bias (Bellotti et al., 2011; Branthwaite & Garcia, 1985).

## Discussion

This study sought to investigate the impact of employment programs on primary and secondary outcomes of common mental disorders. Included in the review were five studies with distinct interventions. Results suggest employment programs may have overall positive, yet mixed effects on severity of CMD across time. About 2 out of 5 (40%) of included studies reported a positive effect of the intervention on CMDs while 60% showed mixed or no effect.

Inconsistent yet generally positive findings raise the question of whether there was an active employment component that had a greater impact on common mental disorders and what role context played in employment program success. Of the interventions with a skills-based training component, 60% found a positive impact on mental health, whereas both studies with a placement component showed null effects, although both studies had various methodological shortcomings (Bellotti et al., 2011; Branthwaite & Garcia, 1985).

The mixed effects in studies with similar active employment components likely reflects the high heterogeneity and different content of programs in included studies. Interventions with skills-based training had different styles of training with many utilizing groups while other employing individual sessions. The number of sessions and length and content of training was also diverse. Whether programs included placements or on-the-job-training also varied. These differences may partially account for mixed results of their impact on CMD.

Study design may also contribute to mixed findings. All RCT studies reported a positive effect though non-randomized experiments, and cohort studies did not. The small sample size and thus inadequate power in two studies reporting null effects likely also played a role in overall mixed effects. Non-randomized and retrospective cohort designs are weaker designs than RCTs, making them more susceptible to confounding. Other study factors may also play a role. For example, the gender focus of interventions did seem to have differential impacts with the study with a mainly female sample reporting a positive impact on CMD and only 1/4 of the studies with a general sample (or a sample of mainly men) reporting an overall positive effect. Greater research on the differential impact of employment programs by gender is needed for any conclusions to be drawn.

There was also substantial heterogeneity in how CMDs were measured within included studies. A variety of scales were used to measure outcomes and all but two studies assessed CMDs after participants were screened and included into the study. Most studies looked more at a decrease in score of the CMDs measured than clinical cut-offs. Having this heterogeneity wherein some studies had clinical samples and others had general samples may also account for some of the mixed effects within study populations. While research has shown that employment programs, particularly vocational rehabilitation and supported employment, positively impact both vocational and mental health outcomes in those with SMI, gaining further insight into the effect on incidence and prevalence of CMD is imperative (McGuire et al., 2020; Modini et al., 2016). At the same time, evaluating the impact of employment programs on CMD outcomes of those in the general population aids in understanding the widespread impact of these programs. In the future, reviews could look at clinical versus general population samples separately as these two populations may be impacted differently by employment programs.

There are several limitations to this review. Firstly, gray literature was not searched or included in analysis. As a result, some relevant articles and materials may have been missed in screening. Second, the search was not complemented by qualitative studies which may have helped in analyzing participants’ reactions to programs and may have provided greater understanding into the mixed effects found in this review and the role of context. The broad nature of the search strategy and inclusion/exclusion criteria also led to high heterogeneity within included studies, making it difficult to form wider claims about the nature of employment programs and their impact on CMD and calculate a pooled estimate.

## Conclusions

This review sought to investigate the impact of employment programs on CMDs, such as anxiety and depression. While results were mixed, over 60% of included studies reported a positive impact on at least one CMD. Employment programs show promise in improving common mental disorder outcomes through reducing poverty and other social determinants of mental ill health. The main recommendation from this review is for an increase in research on employment programs and their impact on CMD. Further research should solidify the nature of the association and additional analysis on the mechanisms of impact and persistence of effect would allow for insight into how these interventions work and why they may lead to decreased CMD prevalence, incidence, or severity.

Current employment programs should focus on communities and sub-populations with greatest need for increased social and economic capital and thus those also most impacted by poverty, unemployment, and CMD (such as women). Due to the possibility of relapse, those with CMD may require mental health support during employment programs to ensure they effectively engage with the programs and impact is sustainable. It is imperative that participants are supported in cases of clinical deterioration to get the proper care to support their wellbeing. Integrating employment programs with other poverty-reduction, social capital, and/or psychosocial interventions may provide sustained effects of psychosocial interventions and improve symptoms of people with CMD in the long run (Pega et al., 2017; Purgato et al., 2018). A focus on holistic prevention and treatment of CMD through poverty reduction, employment, and mental health care programs could enable management of the symptoms of poor mental health while also addressing chronic social determinants of mental ill health.

## Supplemental Material

sj-docx-1-isp-10.1177_00207640221104684 – Supplemental material for The impact of employment programs on common mental disorders: A systematic review Click here for additional data file. Supplemental material, sj-docx-1-isp-10.1177_00207640221104684 for The impact of employment programs on common mental disorders: A systematic review by Libby Evans, Crick Lund, Alessandro Massazza, Hannah Weir and Daniela C Fuhr in International Journal of Social Psychiatry
