# Supplementary material for: The impact of employment programs on common mental disorders: A systematic review
Source: Int J Soc Psychiatry. 2022 Jul 7;68(7):1315–23. doi: 10.1177/00207640221104684 (PMC9548920; doi:10.1177/00207640221104684)
Supplement: sj-docx-1-isp-10.1177_00207640221104684 – Supplemental material for The impact of employment programs on common mental disorders: A systematic review [file sj-docx-1-isp-10.1177_00207640221104684.docx]

**Appendix 1: Search strategy**

1. anxiety OR anxiety/ OR performance anxiety/ OR anxiety disorder/ OR generalized anxiety disorder/
2. depression/ OR common mental disorder OR anxiety disorder
3. neurosis/ OR neurotic
4. stress-related OR mental stress
5. somatoform disorder/ OR somatoform
6. 1 OR 2 OR 3 OR 4 OR 5 OR 6
7. entrepreneur training
8. entrepreneur program*
9. job-match
10. microfinance
11. cash transfer
12. unconditional cash transfer
13. conditional cash transfer
14. micro-credit
15. microcredit
16. “vocational rehab*”
17. “occupation* rehab”
18. “supported employment”
19. apprentic*
20. “work program*”
21. “job placement assistance”
22. “skills building”
23. “cash grant”
24. “public work program*”
25. 8 OR 9 OR 10 OR 11 OR 12 OR 13 OR 14 OR 15 OR 16 OR 17 OR 18 OR 19 OR 20 OR 21 OR 22 OR 23 OR 24 OR 25
26. 7 AND 26

**Appendix 2: Inclusion and exclusion criteria**

|  | Included | Excluded |
| --- | --- | --- |
| Study Design | - Quantitative studies (RCTs, cohort, case control, or cross-sectional). - Mixed-methods studies. - English language studies. | - All other study designs. Studies not published in English. - Qualitative studies. |
| Population | - General population of all ages - HICs and LMICs |  |
| Intervention | - The most comprehensive definition of an employment program is a program with any direct employment creation activities. Employment programs include a wide spectrum of options, varying from the more conventional public works programs (PWPs), such as short-term emergency programs, to employment guarantee programs. Employment programs refer to the more common and traditional programs, which may be temporary responses to speciﬁc shocks, crises, or disabilities such as ISPS and supported employment. But they can also have a longer-term horizon (cash and food for work programs, train-and-place, etc.). (23) For the sake of this review, they do not have to be public work programs but can be implemented by an NGO or other group for these same purposes. | - Psychological or mental health interventions implemented in the workplace - Vocational or occupational rehabilitation programs that don't have an active employment program component. - Psychological or mental health interventions and vocational / occupational rehabilitation programs focusing on mental rehabilitation without an active employment program component will be excluded - Cash transfer, loan interventions, and/or other social protection programs not directly focusing on employment. |
| Outcome | - Measurement of a common mental disorder as a primary or secondary outcome, either through use of a screening or diagnostic tool. This could include a change in depression scores or change in proportion of people diagnosed with or experiencing symptoms of a common mental disorder. | - No measurement of one or more common mental disorder. - Tool/measure validation studies. - Severe mental illness, autism disorders, or alcohol use disorders as a study’s primary outcome. |

**Appendix 3**

*Skills-based training component descriptions*

| **Article reference** | **Skill-based training component** |
| --- | --- |
| Bellotti 2011 | Educational training and courses on conservation skills and green jobs. |
| Branthwaite 1985 | Participants in the project scheme arm of the intervention are trained on a skill of craftsmanship such as bricklaying, carpentry, decorating, or landscaping. |
| Glass 2017 | Family is trained on basic livestock nutrition, care, and basic health services as part of the asset transfer. |
| Karasz 2021 | Group sessions and trainings where participants are taught financial literacy education alongside psychoeducation prior to a cash-transfer. |
| Vinokur 2000 | Training on the job search, motivation, and inoculation to setbacks training done in five, four-hour sessions over the span of a week. |

**Appendix 4: Risk of bias tables**

**Table A**

*Cochrane risk of bias assessment*

|  | | |  |  |  |  |  |
| --- | --- | --- | --- | --- | --- | --- | --- |
| **Reference** | **Random sequence generation** | **Allocation concealment** | **Blinding (participants and personnel)** | **Blinding (outcome assessment)** | **Incomplete outcome data** | **Selective reporitng** | **Other sources of bias** |
| Glass 2017 | Unclear | Unclear | Low risk | Low risk | Unclear | Low risk | Low risk |
| Karasz 2021 | Low risk | Low risk | Low risk | Unclear | Low risk | Low risk | Low risk |
| Vinokur 2000 | Low risk | Unclear | Unclear | Unclear | Low risk | Low risk | Unclear |
|  |  |  |  |  |  |  |  |

**Table B**

*Newcastle-Ottawa Quality rating for cohort studies*

| **Reference** | **Selection** | **.** | **.2** | **.3** | **Comparability** | **Outcome** | **.4** | **.5** | |  |
| --- | --- | --- | --- | --- | --- | --- | --- | --- | --- | --- |
|  | Representativeness | Selection | Ascertainment of exposures | Outcome not present at beginning | Comparability of cohorts | Assessment of outcome | Follow-up length | | Adequacy of follow-up | |
| Branthwaite 1985 |  |  | * |  |  | * | * |  | |  |
| Bellotti 2011 |  |  | * |  |  |  | * |  | |  |
